# Geroprotective interventions converge on gene expression programs of reduced inflammation and restored fatty acid metabolism

**DOI:** 10.1007/s11357-023-00915-1

**Published:** 2023-09-12

**Authors:** Tomer Landsberger, Ido Amit, Uri Alon

**Affiliations:** 1 Department of Systems Immunology, Weizmann Institute of Science, Rehovot, Israel; 2 Department of Molecular Cell Biology, Weizmann Institute of Science, Rehovot, Israel

**Keywords:** Geroprotective interventions, Rejuvenation, Aging, Longevity, Inflammation, Transcriptomics

## Abstract

Understanding the mechanisms of geroprotective interventions is central to aging research. We compare four prominent interventions: senolysis, caloric restriction, in vivo partial reprogramming, and heterochronic parabiosis. Using published mice transcriptomic data, we juxtapose these interventions against normal aging. We find a gene expression program common to all four interventions, in which inflammation is reduced and several metabolic processes, especially fatty acid metabolism, are increased. Normal aging exhibits the inverse of this signature across multiple organs and tissues. A similar inverse signature arises in three chronic inflammation disease models in a non-aging context, suggesting that the shift in metabolism occurs downstream of inflammation. Chronic inflammation is also shown to accelerate transcriptomic age. We conclude that a core mechanism of geroprotective interventions acts through the reduction of inflammation with downstream effects that restore fatty acid metabolism. This supports the notion of directly targeting genes associated with these pathways to mitigate age-related deterioration.

**Supplementary Information:**

The online version contains supplementary material available at 10.1007/s11357-023-00915-1.

## Introduction

Aging is characterized by an accumulation of damage on multiple levels of biological organization that causes progressive loss of function and increased vulnerability to illness, thereby limiting organismal lifespan. Multiple interventions operating via different modalities have been shown to delay and, in some cases, reverse age-related changes, and increase the lifespan of model organisms. Mahmoudi et al*.* 2019 [1] delineated four main interventions for geroprotection and life-extension in mammals: 1. Exchange of blood-borne factors between young and old animals, e.g. through heterochronic parabiosis, a surgical procedure whereby two animals of different ages are joined together such that they share a common circulatory system. As a result of this exchange, young parabiont experience anti-aging effects whereas the old parabiont experience pro-aging effects; 2. Metabolic manipulation, including caloric restriction, the oldest known and most widely applicable life-extending intervention; 3. Ablation of *senescence cells*, which are cells that underwent a stress-induced cell-cycle exit, that accumulate with aging and secrete pro-inflammatory factors; 4. in vivo partial reprogramming, activation of Yamanaka factors in a transient manner to achieve cellular rejuvenation while avoiding induction of a pluripotent state and subsequent teratoma formation.

Despite extensive research into these interventions, and although translation efforts are underway [2], key questions remain unanswered. One such question is what, if any, are the shared effects and mechanisms of action of these disparate interventions. Recently, transcriptomic data pertaining to the four paradigmatic geroprotective interventions (GIs) has become available [3–6], calling for a comprehensive comparative analysis. Directly comparing the gene expression signatures of GIs to one another and to that of normal aging may illuminate core aspects of geroprotection and uncover new targets for therapeutics.

In this study, we utilize published bulk and single-cell RNA-seq (scRNA-seq) datasets from mice corresponding to the four GIs, aging, and inflammatory disease models, to investigate the shared effects and potential modes of action of GIs. We identify several genes that are coordinately affected by GIs across organs and cell types. We find a robust GIs pathway signature that aligns with the aging pathway signature but in reverse. Inflammation and inflammation-related processes, as well as IGF transport and uptake, wound healing, and cell-to-cell adhesion, are downregulated in all GIs and upregulated in aging. Metabolic processes, particularly lipid and fatty and carboxylic acid metabolism, are upregulated in all GIs and downregulated in aging. By analyzing data from chronic inflammation in young mice, we demonstrated that the metabolic changes observed are likely a downstream effect of inflammation. A transcriptomic age calculator trained on human data assigns lower scores to samples from mice subjected to GIs and higher scores to samples from chronic inflammation models, compared to their respective controls, suggesting inflammation to be a strong determinant of transcriptional age in mammals. Overall, this study emphasizes the role of inflammation-related processes in GIs and in normal aging.

## Methods

### Choice of datasets for analysis

GI datasets were chosen based on availability, breadth, and quality. RNA-seq was prioritized over microarray-based datasets (multiple of which exist for caloric restriction) for the sake of consistency. Aging datasets used as references in the analysis of chronic inflammation (CI)  models were taken from *Tabula Muris Senis* [7, 8] due to its comprehensiveness. Droplet data was used for an optimal match with the CI dataset. For the analysis of single-cell aging lungs, the *Angelidis* et al. aging lung cell atlas [9] was used instead of *Tabula Muris Senis* owing to greater cell type overlap with *Strunz* [10] idiopathic pulmonary fibrosis dataset. In reprogramming, spleen samples were excluded due to poor signal.

### Bulk differential gene expression analysis

The standard workflow of DESeq2 [11], which is based on the negative binomial distribution, was used for all bulk datasets. DEGs are selected to have |fold-change|> 1.25 and Benjamini–Hochberg adjusted p-value < 0.05. Tests were controlled for sex as a covariant. For *Tabula Muris Senis* data, 24- and 27-month-old mice were compared to 3-month-old mice. For caloric restriction, DEG analysis results provided in the original publication were used.

### Single-cell clustering and cell-population annotation

To facilitate a direct comparison of CI models to aging from independent studies, some of the annotations that appeared in the original publications needed to be re-established so that cell types can be accurately matched (Supplementary Fig. 1). Cell type annotations were adopted from original studies when applicable (lung: *Strunz/Angelidis*, kidney: *Tabula Muris Senis*). Where published annotation was deemed too coarse-grained (lung: *Tabula Muris Senis*, liver: *Xiong*/*Tabula Muris Senis*, kidney: *Conway*), Seurat package (version 4.0) [12] standard workflow was used for clustering. Briefly, cells with > 15% mitochondrial content were removed, the UMI matrix was log-normalized, highly variable genes were detected (following exclusion of mitochondrial, ribosomal and cell-cycle genes), and PCA was applied, followed by KNN graph construction on 50 leading components. The Louvain algorithm was used for community detection using resolution parameter 1.5. Clusters were manually annotated based on marker genes and cross-reference with the pre-annotated dataset. Not all mice used for clustering are used for downstream analysis.

### Single-cell ambient RNA removal

Ambient RNA is RNA present in the cell suspension that can be aberrantly counted along with a cell’s native mRNA. In most datasets, ubiquitous expression of highly-expressed cell-type specific genes (e.g., surfactant genes in lung tissue) suggested such cross-contamination. To overcome this, the decontX() function from celda (V1.6.1) [13] was used with default parameters to remove contamination in individual cells. decontX is a Bayesian method that models the empirical expression of a cell as a mixture of counts from two multinomial distributions: (1) a distribution of native transcript counts from the cell’s actual population and (2) a distribution of contaminating transcript counts from all other cell populations captured in the assay.

### Single-cell differential gene expression analysis

For heterochronic parabiosis data, DEGs were adopted from the original study. For CI models data, and *Tabula Muris Senis*-derived aging reference, DEGs were re-derived, following clustering and annotations (as per single-cell clustering and cell-population annotation), using a two-sided MAST [14] test implemented in Seurat [12] V4. MAST analysis was applied to decontaminated log-transformed normalized counts for each group and each cell type individually. Genes were pre-filtered to those expressed in > 0.01 of cells and with > 5 cells with > 1 UMIs. DEGs are selected to have |fold-change|> 1.25 and Benjamini–Hochberg adjusted p-value < 0.05. Tests were controlled for sex as a covariant.

To avoid ambient RNA, in addition to using DecontX corrected data, we also excluded DEGs if they met both of these criteria: 1. among the top 5 markers in another cell type *and* 2. among the top 50 most highly expressed genes in the dataset.

We note that for *Strunz* et al. lung fibrosis study, which performed time-course measurements, we used cells from 7 days post-bleomycin treatment onwards.

For liver and kidney from *Tabula Muris Senis*, tests were applied for aged vs. young, where “aged” consists of cells pooled from 18-, 21-, 24-, and 30-month-old mice, and “young” consisted of 3-month-old mice.

### Gene set over-representation analysis

MetaScape [15] web interface for multiple gene lists was used to analyze DEGs with parameters: Min Overlap = 2, *P*-Value Cutoff = 0.01, and Min Enrichment = 1.5.

### Pathway scoring in GI/aging

Each whole organ-related hit (FDR < 0.01) contributed 1 point, and each cell type-related hit from the heterochronic parabiosis study contributed 0.5 points to the total rank score.

### Correlation analysis

We performed pairwise Spearman correlations of the fold-changes of shared DEGs for each pair. When the DEG set was < 10, the pair was excluded. Only correlations where p-value < 0.05 are presented in the heatmaps.

### Application of RNAAgeCalc gerometer

RNA age was calculated using RNAAgeCalc [16] trained on human data derived from GTEx V6 to construct a cross-tissue and tissue-specific transcriptional age calculator. The algorithm was implemented in R following the guidelines described in https://www.bioconductor.org/packages/release/bioc/vignettes/RNAAgeCalc/inst/doc/RNAAge-vignette.html, with tissue-specific predictor used when applicable and pan-tissue predictor used for liver and kidney and muscle. The counts matrix was supplied. When counts were UMIs (ABT), gene lengths were set to 10 K for all genes. Mouse gene names were converted to human homologs using convert_mouse_to_human_symbols() function from the nichenetr [17] library in R. The readout, given in human years, was scaled for presentation.

### Visualization

Plots were generated in R using ggplot2 [18] and ComplexHeatmap [19] R libraries.

## Results

### Geroprotective interventions share gene expression and pathway signatures that are inverse to normal aging

**Fig. 1:**
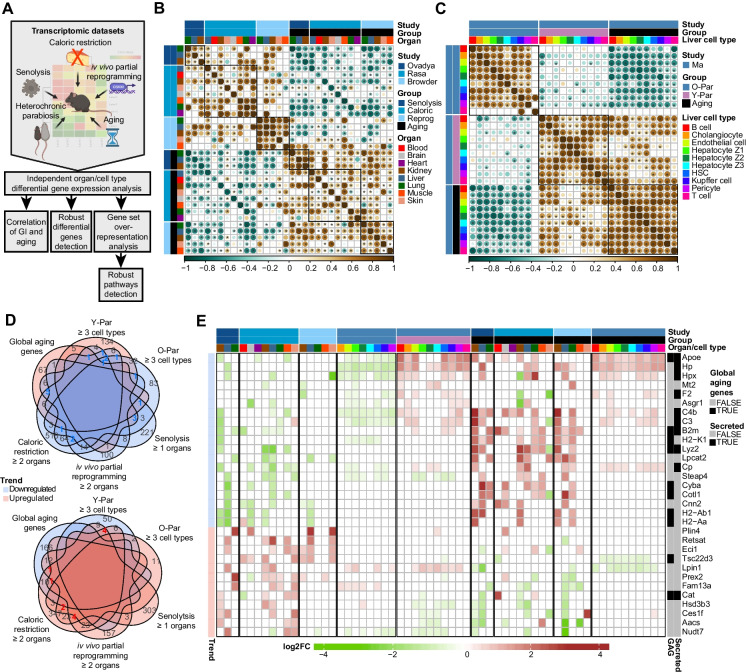
Geroproective interventions share a gene expression signature which is inverse to aging: **A** Flowchart describing the data and analysis. **B** Mutual Spearman correlations of gene expression signatures of senolysis, reprogramming, caloric restriction, and aging from different organs (bulk). Only pair-wise correlations where shared DEG set > 10 are calculated and colored. Correlation coefficient is encoded by both color and circle size. Asterisks depict p-value < 0.05. Color annotated for study, group, and organ. **C** Same as B but for heterochronic parabiosis and aging from different cell types (single-cell). **D** Venn diagram of downregulated DEGs from senolysis (≥ 1 organ), caloric restriction (≥ 2 organs), reprogramming (≥ 2 organs), heterochronic parabiosis (≥ 3 cell types); upregulated DEGs from Y-Par (≥ 3 cell types), and upregulated global aging genes (top), and of the opposite trend (bottom). DEGs selected to have (|fold-change|> 1.25, FDR < 0.05). Labeled for number of DEGs, with those shared for 3 or more sets highlighted. Color-coded for up/downregulation. **E** heatmap depicting DEGs that are shared by 3 or more sets. Color-coded for log2 expression fold-change (clipped at -/ + 4). Color annotated for study, group, and organ/cell type (top); for trend (left), for belonging to global aging genes set, and for coding for secreted factor (right). Abbrev: Par = parabiont; Y = young; O = old; DEG = differentially expressed gene

To identify common modes of action of geroprotective interventions (GIs), we sought to compare the transcriptomic changes associated with the four GIs, to each other and the transcriptomic changes associated with normal aging. To that end, we analyzed bulk and scRNA-seq data from four independent studies that tested these interventions in mice (Supplementary Table 1): 1. Ablation of senescent cells, using the senolytic agent ABT-737 [3]. 2. Long-term caloric restriction [4], decreased calorie intake without malnutrition starting from 4 months of age. 3. In vivo partial reprogramming (hereinafter *reprogramming*) [5], using transgenic mice carrying a single copy of a polycistronic cassette which enables the systemic expression of OSKM by administering doxycycline. 4. Heterochronic parabiosis [6], studied in multiple organs (of which we used liver-derived data) at single-cell resolution. Each study also included a young control allowing a direct comparison of the GI to aging. The heterochronic parabiosis study included, in addition to the heterochronic pair, also old and young isochronic pairs as controls. We used these datasets to performe a comparative analysis, as outlined in Fig. 1A.

Firstly, we re-derived senolysis and reprogramming-related differential expressed genes (DEGs) by comparing treated animals to their respective aged controls. Aged controls were also compared to young controls to generate intra-study aging reference signatures (methods, Supplementary Table 2). Next, we correlated the gene expression fold-changes (hereafter *signatures*) associated with GIs in different organs with one another, and with the signatures associated with aging (methods). We performed this analysis separately for bulk and single-cell (heterochronic parabiosis) data.

We found that for most organs and interventions, the GI signatures analyzed at bulk-level exhibit mutual (Spearman) correlations in a pan-organ and pan-intervention manner, and are anti-correlated with the aging signatures, which also show mutual correlations in a pan-organ and pan-study manner (Fig. 1B). For heterochronic parabiosis, analyzed at a single-cell level, we find that the signatures of heterochronic vs isochronic old parabionts (O-Par) are mostly correlated in a pan-cell-type manner, and mostly anti-correlated with both heterochronic vs isochronic young parabionts (Y-Par) and with isochronic old vs young (aging) signatures. Consistent with that, Y-Par and aging signatures are mostly mutually correlated in a pan-cell-type manner (Fig. 1C). We thus conclude that GIs partially reverse the aging signature in a similar fashion and that Y-Par partially recapitulates the aging signature.

We aimed to identify the genes that are consistently influenced by GIs and inversely affected by Y-Par and aging by intersecting their associated DEG sets. DEG sets for GIs were derived such that they appear in at least 1 organ in senolysis, at least 2 organs in reprogramming and caloric restriction, and at least 3 cell types in heterochronic parabiosis. The aging-associated DEGs were obtained directly from a study [20] that methodically analyzed the *Tabula Muris Senis* [7], a comprehensive age-resolved murine bulk and single-cell transcriptomic dataset, to identify global aging genes. The identified global aging genes consist of 93 upregulated and 190 downregulated genes.

Considering the correlations described in Fig. 1B and C, we intersected upregulated DEGs associated with GIs with downregulated DEGs associated with Y-Par and downregulated global aging genes, and vice versa (*trends*, Fig. 1D). While most DEGs are set-specific, some are shared by up to 5 sets.

DEGs that are shared between 3 or more sets are depicted in a heatmap (Fig. 1E). The GI-downregulated genes consist mainly of immune-related genes. This demonstrates that GIs reduce the low-grade inflammatory phenotype pervasive in mammalian aging (i.e., *inflammaging*). This set includes genes encoding for components of MHC class-I (*B2m*, *H2-K1*) and Class-II (*H2-Ab1*, *H2-Aa*), complement system (*C3*, *C4b*), and lysozyme (*Lyz2*). We also find *Apoe*, the gene whose polymorphisms are associated with human longevity [21, 22]. Many of these genes encode for secreted factors, underscoring their potential systemic effect.

The GI-upregulated genes comprise a less functionally cohesive group. Intriguingly, *Cat*, which encodes for Catalase, is upregulated in some of the studied organs in both senolysis and caloric restriction and is also a downregulated global aging gene. Catalase is a key antioxidant enzyme that defends against oxidative stress and has been linked to age-related diseases [23]. Its mitochondrial [24] and cardiac [25] -targeted overexpression has a life-extending effect in mice. *Lpin1*, *Tsc22d3,* and *Plin4* appear in the GenDR database [26], which catalogs genes whose expression is associated with caloric restriction. None of the other downregulated genes have previously been connected to GIs or aging, according to the Human Aging Genomic Resources [27].

**Fig. 2:**
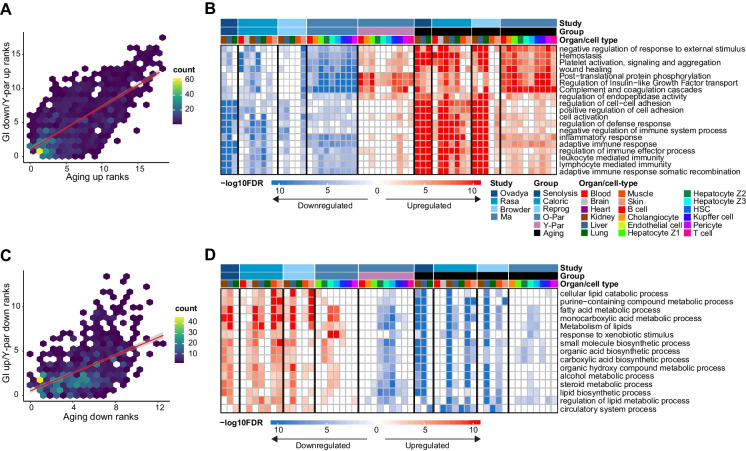
Geroproective interventions share a pathway signature which is inverse to aging: **A** aging upregulated pathways rank scores (x-axis) vs. GI downregulated/Y-Par upregulated pathways rank scores (y-axis). **B** heatmap depicting top scoring pathways for GI down/Y-Par up, alongside aging. Color-coded for -log10 FDR (clipped at 10), with blue downregulated and red upregulated, showing only FDR < 0.01. Color annotated for study, group, and organ/cell type. **C** same as A but for the opposite trend. **D** same as B. for the opposite trend. Abbrev: Par = parabiont; Y = young; O = old; GI = geroprotective intervention

To characterize the shared biological processes that are impacted by the different GIs, we applied gene set over-representation analysis by MetaScape [15] to DEG sets derived from GIs, Y-Par, and aging in the different organs and cell types. First, we co-analyzed downregulated GI DEGs with upregulated Y-Par and aging DEGs. We scored each pathway based on the number of tested DEG sets where it is enriched (methods). We found that the IGs/Y-Par rank scores are correlated with aging rank scores, and particularly that the highest scoring pathways for IGs/Y-Par are also the highest scoring pathways for aging (Fig. 2A). This indicates that the shared GIs downregulated pathways signature aligns with the aging upregulated pathways signature.

Some of the most robust shared pathways were inflammation-related, including complement system and adaptive immune responses (Fig. 2B, Supplementary Table 3), consistent with the gene-level analysis. Inflammation has been identified as a gene expression hallmark of aging across various organs [28] and mammalian species [29]. Concerning GIs, *Mahmoudi *et al*.*[1] concluded that inflammation is mitigated in caloric restriction and heterochronic parabiosis, but that only indirect evidence exists to support its mitigation in senolysis and reprogramming. Exercise was also reported to ameliorate age-associated inflammation [30]. Here we observe the shared mitigation of inflammation by the four GIs.

Other prominent shared pathways include wound healing, cell-to-cell adhesion, and regulation of Insulin − like Growth Factor (IGF), the latter of which is closely linked to aging, and its inhibition in adult or late life confers life-extension in female mice [31]. Lower scoring pathways also included neutrophil degranulation, ERK1/2/MAPK cascade, phagocytosis, and ECM organization (Supplementary Table 3).

We applied the same analysis but this time to DEG sets that are upregulated in GIs and downregulated in Y-Par and aging (i.e., the reverse trend). Here too a correlation exists between IGs/Y-Par and aging-related pathways (Fig. 2C). The pathways most strongly associated with these sets are metabolism-related, especially lipid, and fatty and carboxylic acid metabolism (Fig. 2D, Supplementary Table 3). This aligns with diverse evidence suggesting that lipid metabolism is an important regulator of aging in nematodes, fruit flies, mice, and rats [32]. Moreover, several other studies conducting transcriptomic analysis have reported changes in these pathways in the context of aging [33] (reduced), cellular senescence [34, 35] (enhanced), heterochronic parabiosis [36] (enhanced), and especially in the context of caloric restriction [28, p.], [37–39] (enhanced), where it is foreseeable. Recently, the senolytic agents dasatinib and quercetin were shown to have similar effects on both inflammation and metabolism in adipose tissue, using targeted assays [40].

Our analysis shows that a reduction of inflammation and an increased fatty acid metabolism occurs in all GIs, suggesting a potential mechanistic link between these processes. To investigate this in a non-aging context, we sought to study the transcriptomic signature of inflammation in young animals.

### Chronic inflammation-eliciting disease models in young mice suggests that metabolic changes are downstream of inflammation

**Fig. 3:**
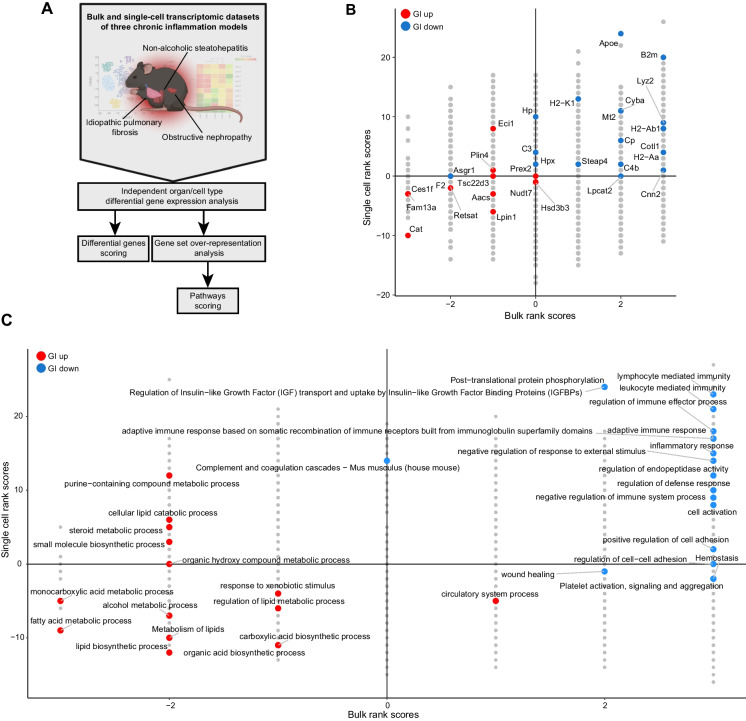
Metabolic changes reversed by geroprotective interventions are downstream of inflammation: **A** Flowchart describing the data and analysis. **B** 2-dimensional map of CI-related DEG rank scores in bulk (x-axis) and single-cell (y-axis) levels. Score is calculated by the number of organs (bulk) or cell types (single-cell) where a gene is a DEG (|fold-change|> 1.25, FDR < 0.05). Upregulation scores 1 point and downregulation scores -1 points. GI-related DEGs from Fig. 1 E are highlighted in color, per their trend in GI. **C** 2-dimensional map of CI-related pathway enrichment rank scores in bulk (x-axis) and single-cell (y-axis) levels. Upregulation scores 1 point and downregulation scores -1 points. GI-related pathways from Fig. 2 B and D are highlighted in color, per their trend in GI. Abbrev: GI = geroprotective intervention; CI = chronic inflammation

To better understand the connection between inflammation and the observed metabolic changes, we turned our attention to bulk and scRNA-seq data derived from murine models of three diseases that involve chronic inflammation, implemented in *young animals* (hereinafter referred to as CI models, Supplementary Table 4): 1. Idiopathic pulmonary fibrosis, bulk [41] and single-cell [10]. 2. Non-alcoholic steatohepatitis, bulk [42] and single-cell [43]. 3. Obstructive nephropathy, bulk [44], and single-cell [45]. We performed the analysis outlined in Fig. 3A.

For bulk datasets, we re-derived DEGs using DESeq2. For single-cell datasets, we re-derived DEGs and in some cases also the cell clustering and annotation, using Seurat [12] package suite and MAST algorithm [14] for the detection of DEGs (methods, Supplementary table 2, Supplementary Fig. 1).

Next, we scored each gene according to the number of times it is significantly upregulated (FC > 1.25, FDR < 0.05) in an organ or cell type in the CI data, minus the number of times it is downregulated (FC < -1.25, FDR < 0.05). We highlighted the GI-shared DEGs presented in Fig. 1E on that map of CI-scored DEGs (Fig. 3B). We found that many of the GI DEGs are differentially expressed also in CI across several organs and cell types, but in the opposite direction. Most genes change in a consistent direction across organs and cell types (Supplementary Fig. 2A).

*B2m* was the most robust upregulated gene, and *Cat* was the most robust downregulated gene. The downregulation of *Cat*, *Fam13a*, and *Ces1f* in all three CI models suggests that these changes occur downstream of inflammation. Hence, their downregulation in aging is likely to be influenced by the presence of inflammation, and their upregulation in GIs may be a downstream effect of inflammation mitigation. However, a reciprocal relationship could exist, where e.g., reduced Catalase may cause increased H2O2-mediated oxidative stress and potentially promote inflammation, and vice versa [46].

We used MetaScape [15] to analyze the DEG sets associated with CI models in different organs and cell types. We scored each pathway in the same way as for genes, counting enrichment (FDR < 0.01) in a given set of DEGs instead of differential expression. We highlighted in color the GI-associated pathways from Fig. 2B and D on the CI-scored enriched pathways map. Inflammation-related pathways are predictably upregulated (Fig. 3C, Supplementary Table 3). Wound healing and cell-to-cell adhesion are upregulated only at the bulk level. Interestingly, metabolism-related pathways are robustly downregulated, with fatty acid metabolism being the most consistently downregulated pathway across CI models. These scores contain contributions from immune cell types as well as parenchymal and stromal cells in all three organs (Supplementary Table 3).

 To further investigate the similarity between CI and aging, we sought to pairwise correlate their gene expression signatures, similar to the analysis of GI and aging. For this purpose, we derived aging signatures from the *Tabula muris senis* [7, 8]*,* separately for bulk and single-cell data (Supplementary Table 5). After matching cell types for CI and aging (Supplementary Fig. 1A-F, Methods), we observed extensive pan-organ and pan-cell type correlations (Supplementary Fig. 1G), where the correlated cell types are from multiple lineages. This shows that the three CI models analyzed partially recapitulate the gene expression signature of aging.

Overall, our findings reveal a consistent inverse relationship between inflammation signals and fatty acid metabolism across three distinct contexts—GIs, aging, and CI. This pattern suggests a mechanistic connection between these processes. Inflammation induces an oxidative burst, leading to oxidative stress [47, 48]. This oxidative stress may cause aberrations in lipid metabolism [49]. Moreover, immune cells undergoing inflammation often experience metabolic reprogramming, characterized by a shift towards glycolysis, which could reduce fatty acid oxidation [50]. This metabolic shift likely serves the dual purpose of meeting the energetic and biosynthetic demands of activated immune cells and also modulating their effector functions.

While our findings suggest that reduced fatty acid metabolism may occur downstream of inflammation, it's plausible that these two processes exist in a reciprocal relationship, dynamically influencing each other. 

### Transcriptional age is increased by inflammation

Several studies have reported the accelerating or decelerating effect of different interventions on so-called *biological age*, as measured by multiparametric *gerometers* (aging clocks), most commonly DNA methylation markers [51]. We hypothesized that inflammation-related signals, comprising the main shared component of both GIs and aging gene expression signatures, are strong determinants of biological age as measured by these methods. To test this, we used RNAAgeCalc [16], calibrated on human data (whole organ bulk RNA-seq) curated in the GTEx [52] data repository. Mouse gene names were converted to their human homologs (methods) to enable this cross-species application, and the age readout was scaled.

**Fig. 4:**
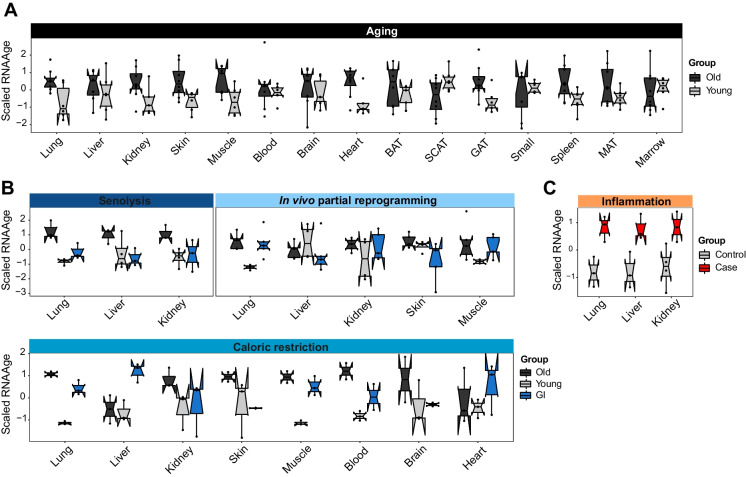
Transcriptional age is increased by chronic inflammation: **A **Scaled RNA age (y-axis) as calculated by RNAAgeCalc for *Tabula Muris Senis*-derived bulk gene expression profiles of old (24/27 months old) and young (3 months old) mice in different organs (x-axis). **B** Scaled RNA age (y-axis) for senolysis, caloric restriction, and reprogramming bulk gene expression profiles in different organs (x-axis). **C** Scaled RNA age (y-axis) for lung (idiopathic pulmonary fibrosis), liver (non-alcoholic steatohepatitis), and kidney (obstructive nephropathy) bulk gene expression profiles. Points represent samples in all panels. Boxplots depict quartiles. Color-coded by group. Abbrev: GI = geroprotective intervention

We find that RNAAgeCalc assigns higher RNA age to samples from old (24/27 months) compared to young (3 months) mice from *Tabula Muris Senis* (Fig. 4A), confirming its applicability to mice data. Furthermore, in many cases, GIs reduce RNA age compared to aged controls (Fig. 4B). Reprogramming reduces RNA age to a significant level only in skin (Fig. 4B), consistent with the original study reporting significant reduction in DNA methylation exclusively in that tissue [5].

Most importantly, in agreement with our hypothesis, RNAAgeCalc assigns higher RNA age to samples from inflamed organs compared to their respective controls (Fig. 4C). Altogether, this demonstrates that inflammation-related genes constitute a mammalian aging biomarker that potentially underlies multiparametric gerometers.

## Discussion

Recent years have seen a surge in attention given to geroprotective interventions (GIs) [1]. Despite the increased focus, our understanding of the mechanisms and effects of GIs remains limited, calling for further exploration. In this study, we reveal a shared feature among the four paradigmatic GIs: a reduction in inflammation. This decrease in inflammation also leads to the restoration of fatty acid metabolism, which is diminished during normal aging. While the role of chronic inflammation in aging is well-known [28, 53, 54], its role in GIs has been less widely acknowledged. Our findings suggest that inflammation and related metabolic changes should be considered hallmarks of not only aging [53] but also GIs.

Our understanding of the aging process implies that changes in gene expression as a result of aging are a combination of passive, aging-promoting changes caused by a drift in the transcriptional landscape and active, aging-antagonizing changes brought on by stress response mechanisms aimed to counteract various damage types incurred to the organism and restore homeostasis. Although these two classes of changes can be difficult to separate, we generally expect the latter to be more coordinated and coherent than the former. Inflammation is a clear example of a programmed response meant to counteract damage to the organism, but it can become deleterious if it persists over time. The metabolic changes we observed in our study may be a less-known result of this harmful overshooting of inflammation. Alleviating inflammaging and restoring proper metabolism could be one way in which GIs exert their anti-aging effects.

GIs may alleviate inflammaging directly, or indirectly by removing the damage signals that cause it. Senolysis eliminates senescent cells thereby reducing SASP (senescence-associated secretory phenotype) that involves inflammatory signals [55]; Caloric restriction curbs inflammation by reducing chromatin accessibility of an inflammation-associated genetic network [4] and may reduce senescent cell accumulation [56]. Cellular reprogramming has been shown to have a rejuvenating effect both ex vivo [57] and in vivo [58], which could involve deactivating stress signals from damaged cells. Lastly, heterochronic parabiosis may reduce systemic inflammation by diluting pro-inflammatory mediators in old blood or introducing youthful anti-inflammatory factors, as well as by reducing senescent cell burden in the old parabiont [59, 60]. It has been shown that a senolytic treatment of old donors reduced pro-aging effects in young recipients after blood exchange [61].

Chronic inflammation in three young mice models led to signatures that align with aging in mice, including reduced fatty acid metabolism. It would be fascinating to further explore the extenet to which chronic inflammation in the young phenocopies aging.

 The opposite effects that CI models and GIs have on transcriptional age are aligned with reported effects on DNA methylation age [51]. Studies have shown that inflammatory conditions such as Covid-19 infection [62] and other stressors [63] can accelerate DNA methylation age, whereas lifestyle changes [64], plasma fraction treatment [65], cellular reprogramming [66], heterochronic parabiosis [36], or drug cocktail [67], can reduce it. Evidence also suggests that even a transient inflammatory stimulus can accelerate DNA methylation age and impair self-renewal in hematopoietic stem cells [68]. These findings, in conjunction with our results, raise the possibility that inflammatory signals contribute significantly to readings of widely used multiparametric gerometers. This may be due to strong signals of inflammatory processes, compared to potentially more subtle and diffuse, yet also more stable, age-related signals. Further research is needed to understand the role of inflammation and downstream signaling in these readings.

In conclusion, our analysis points to inflammation and reduced fatty acid metabolism as robust hallmarks of aging that are reversed by the four paradigmatic GIs. A possible interpretation of our findings is that these two hallmarks are important mediators of the health benefits of GIs. This would argue in favor of directly targeting inflammation and fatty acid metabolism pathways to mitigate age-related health decline.

### Supplementary Information

Below is the link to the electronic supplementary material.

Supplementary file1 (PDF 3.72 MB)

Supplementary file2 (PDF 742 KB)

Supplementary file3 (JPG 902 KB)

Supplementary file4 (XLSX 67381 KB)

Supplementary file5 (XLSX 16218 KB)

Supplementary file6 (JPG 869 KB)

Supplementary file7 (JPG 693 KB)
