# Supplementary material for: Geroprotective interventions converge on gene expression programs of reduced inflammation and restored fatty acid metabolism
Source: GeroScience. 2023 Sep 12;46(2):1627–39. doi: 10.1007/s11357-023-00915-1 (PMC10828297; doi:10.1007/s11357-023-00915-1)
Supplement: Supplementary file 1 — Supplementary file1 (PDF 3.72 MB) [file 11357_2023_915_MOESM1_ESM.pdf]

Supplementary Figure 1 - Correlation of chronic inflammation and aging gene expression signatures

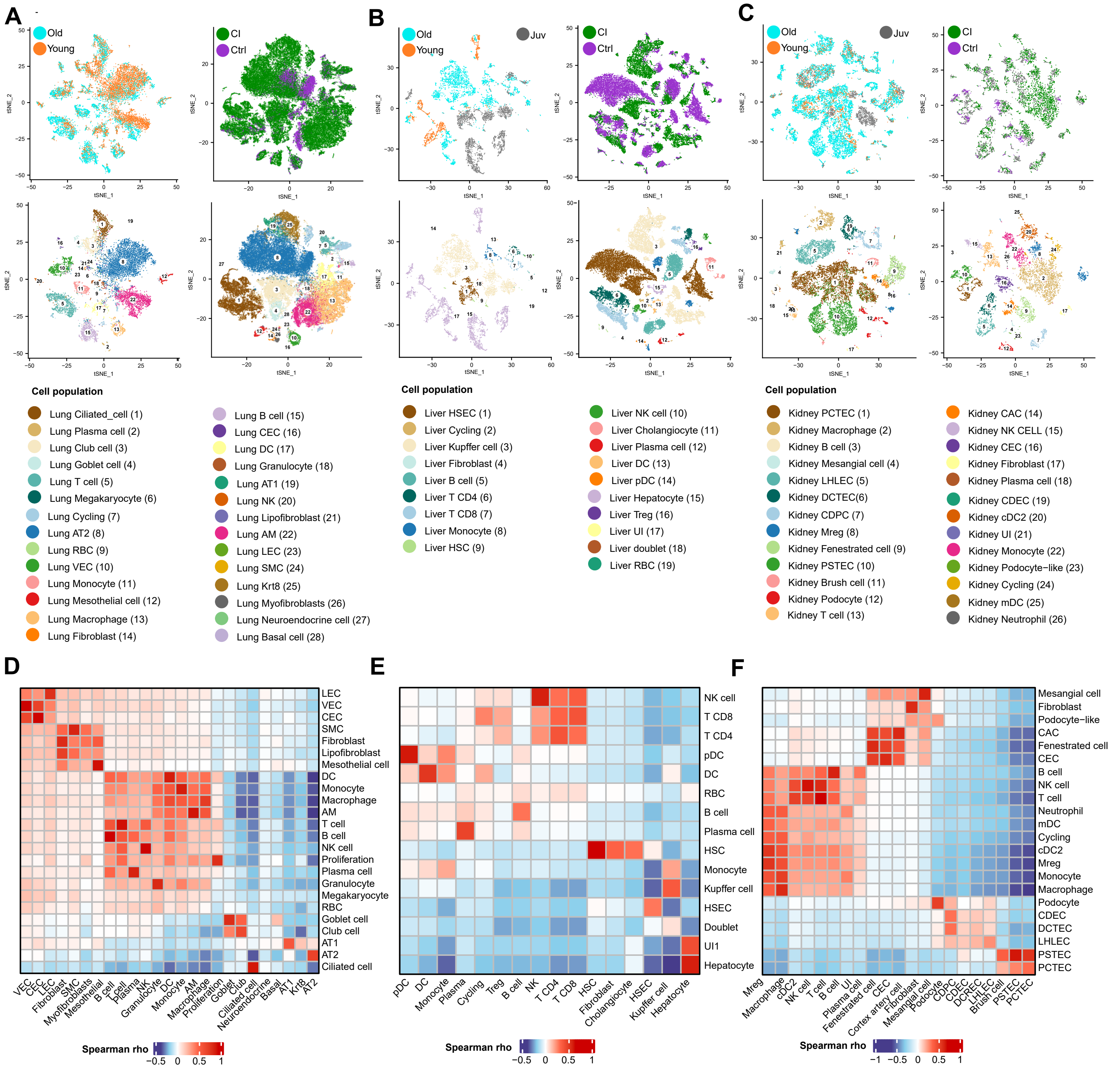

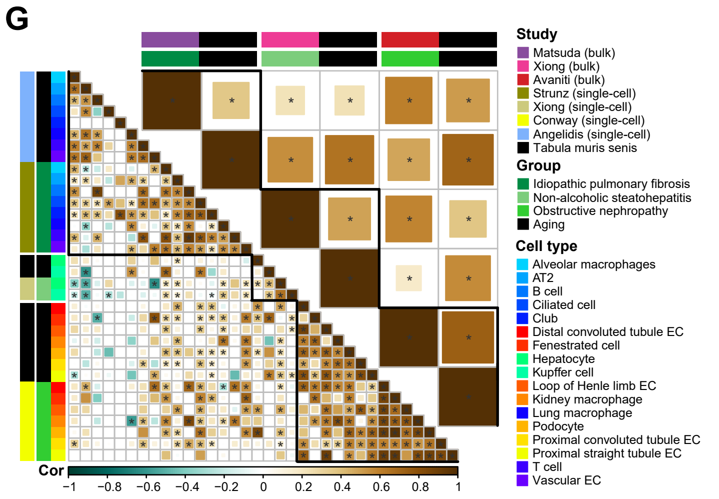

Supplementary Figure 1 - Correlation of chronic inflammation and aging gene expression signatures
A-C, t-distributed stochastic neighbor embedding (tSNE) maps of single cells from aging (left) and CI (right) studies analyzed, color coded for experimental group (top) and for cell population (bottom), in lung (A), liver (B) and kidney (C). **D-F**, Inter-study Spearman correlation per cell population of top 3000 most variable genes in lung (D), liver (E) and kidney (F). **G**, Mutual Spearman correlation of gene expression signatures for CI models and organ-matched aging, in bulk (top) and single-cell (bottom) levels. Only pair-wise correlations where shared DEG set > 10 are colored. Correlation coefficient encoded by both color and square size. Asterisks depict p-value < 0.05. Color annotated for study, group and cell type.
