# Supplementary material for: Geroprotective interventions converge on gene expression programs of reduced inflammation and restored fatty acid metabolism
Source: GeroScience. 2023 Sep 12;46(2):1627–39. doi: 10.1007/s11357-023-00915-1 (PMC10828297; doi:10.1007/s11357-023-00915-1)
Supplement: Supplementary file 2 — Supplementary file2 (PDF 742 KB) [file 11357_2023_915_MOESM2_ESM.pdf]

Supplementary Figure 2 - Gene and pathway analysis for chronic inflammation

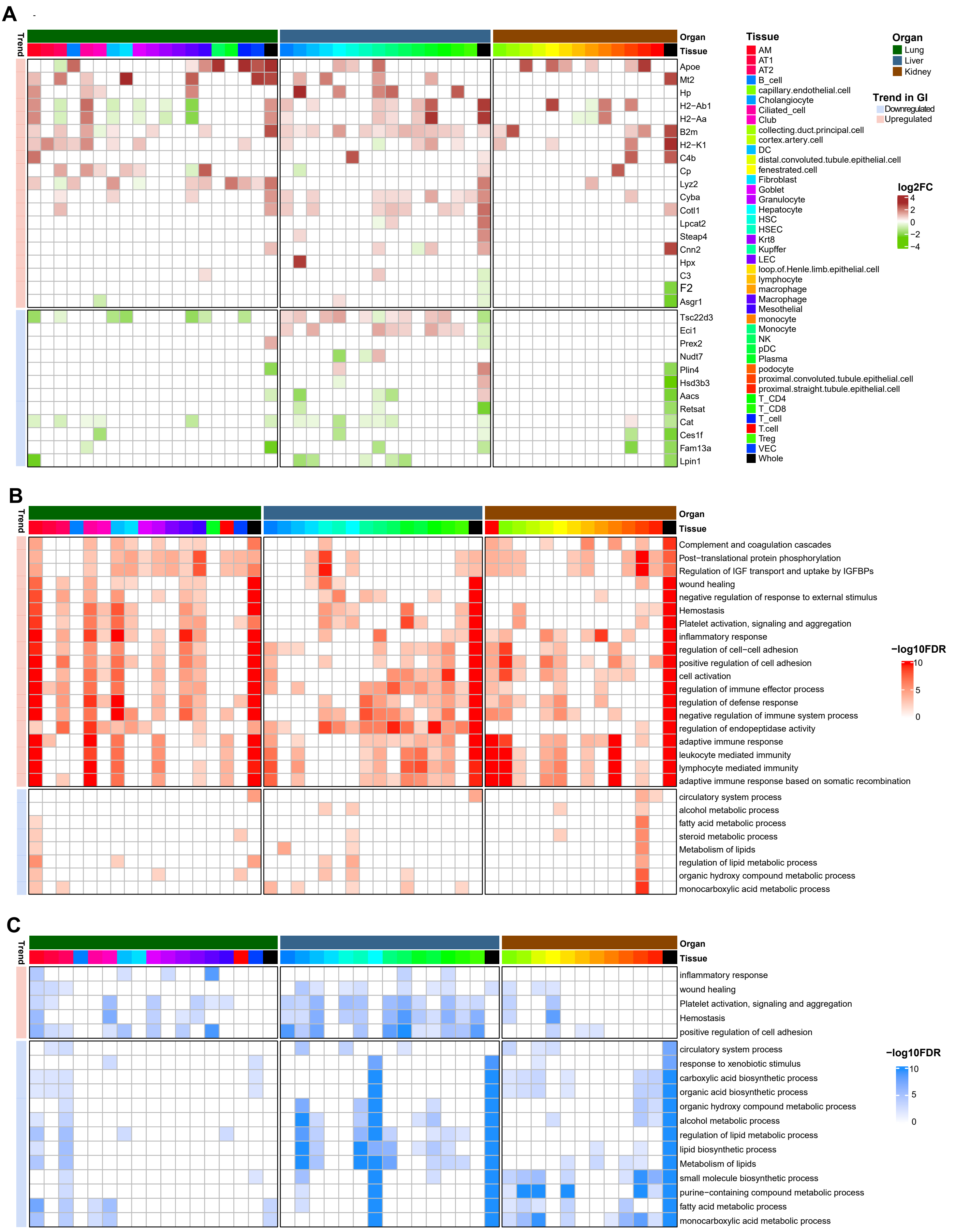

Supplementary Figure 2 - Gene and pathway analysis for chronic inflammation
A, Heatmap depicting top DEGs from figure 1 E. as they change in different organs and cell types in CI. Color-coded for log2 expression fold-change (clipped at -/+4). Color annotated for study, group, and organ/cell type (top); for trend (left), **B.** Heatmap depicting top scoring pathways for from figure 2 B that are upregulated in different organs and cell types in CI. Color-coded for -log10 FDR (clipped at 10), showing only FDR < 0.01. Color annotated for study, group, and organ/-cell type. **C.** same as B but for pathways that are downregulated in CI.
