# Supplementary figures and images for: Geroprotective interventions converge on gene expression programs of reduced inflammation and restored fatty acid metabolism
Source: GeroScience. 2023 Sep 12;46(2):1627–39. doi: 10.1007/s11357-023-00915-1 (PMC10828297; doi:10.1007/s11357-023-00915-1)

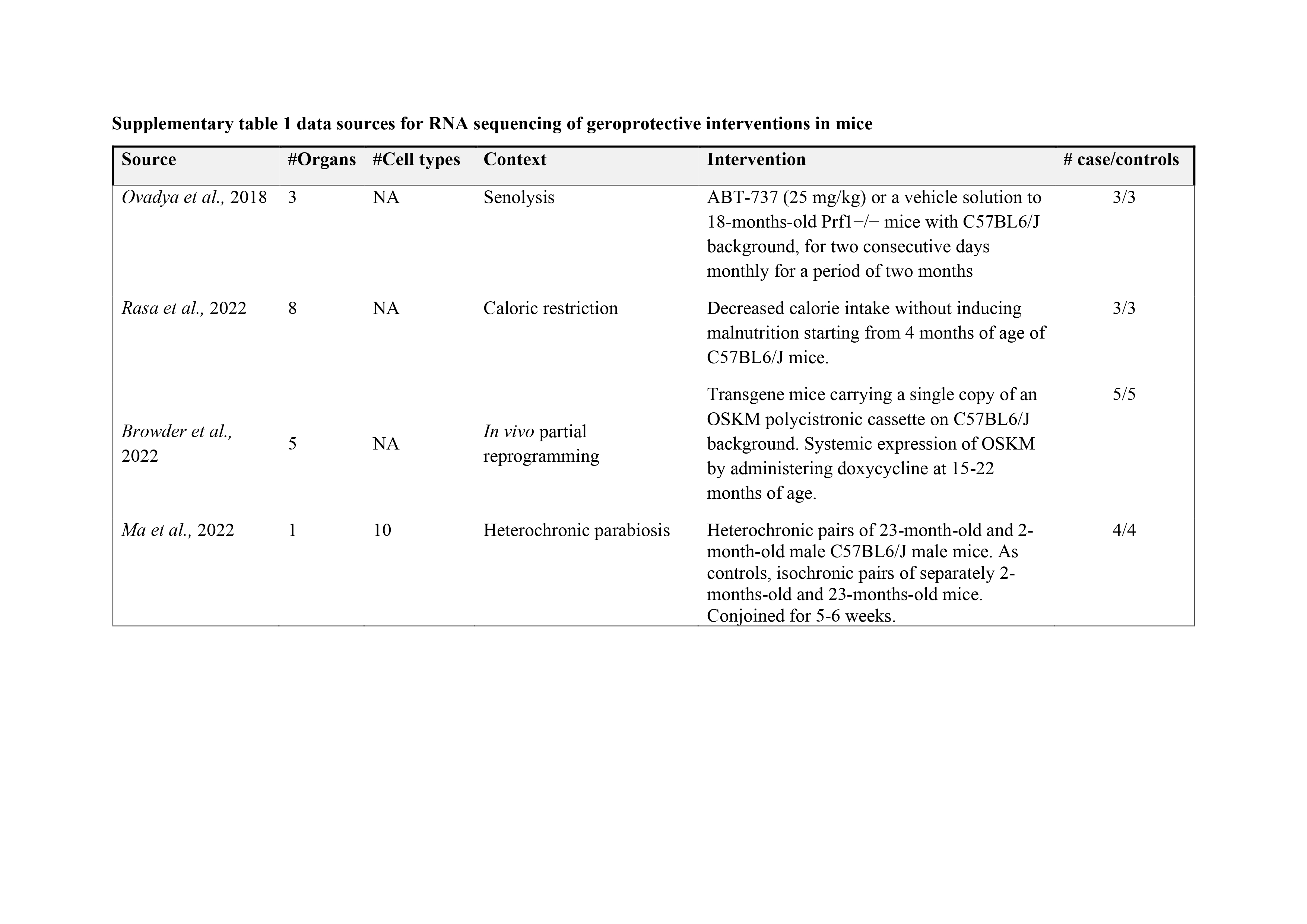

Supplement: Supplementary file 3 — Supplementary file3 (JPG 902 KB) [file 11357_2023_915_MOESM3_ESM.jpg]

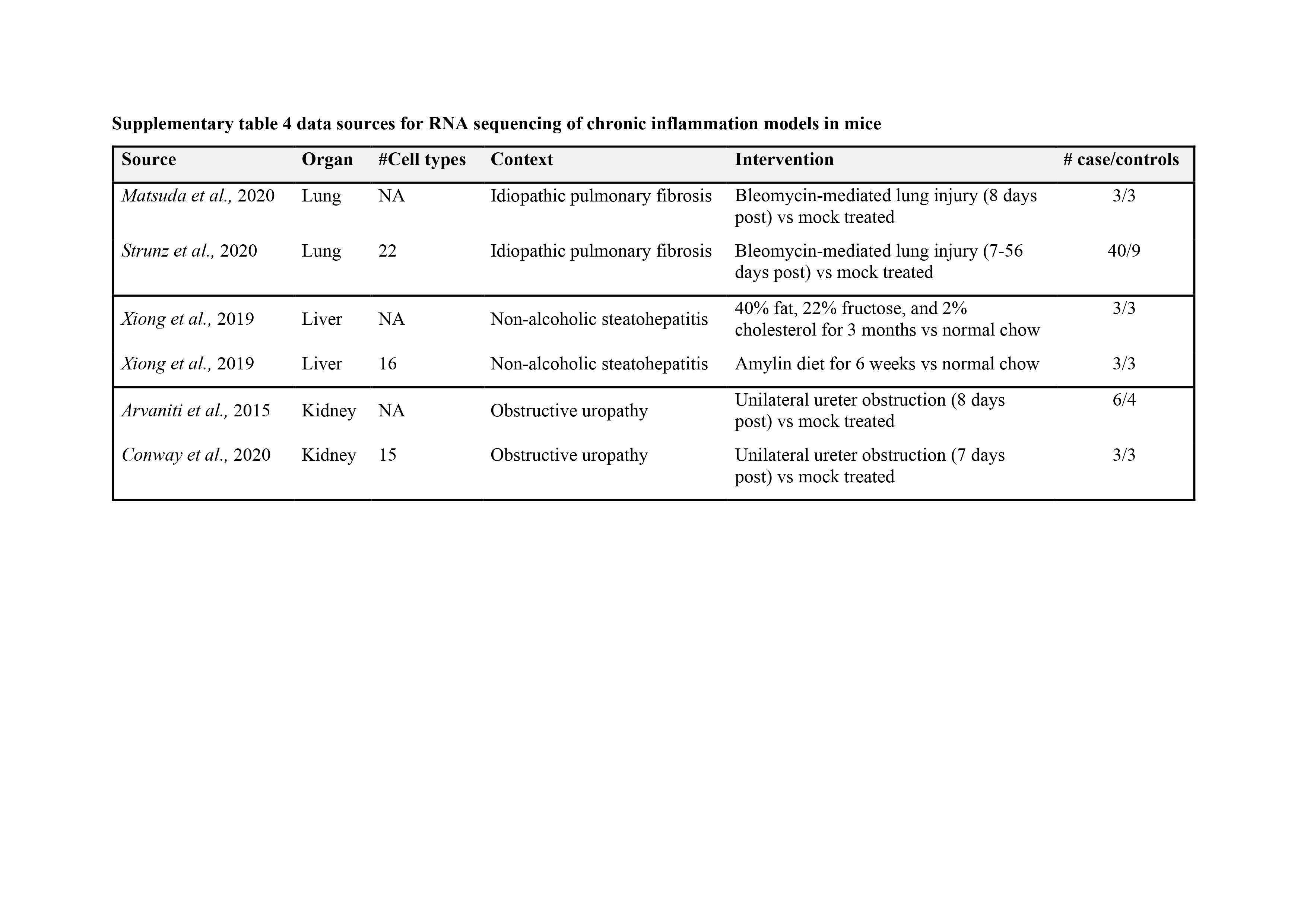

Supplement: Supplementary file 6 — Supplementary file6 (JPG 869 KB) [file 11357_2023_915_MOESM6_ESM.jpg]

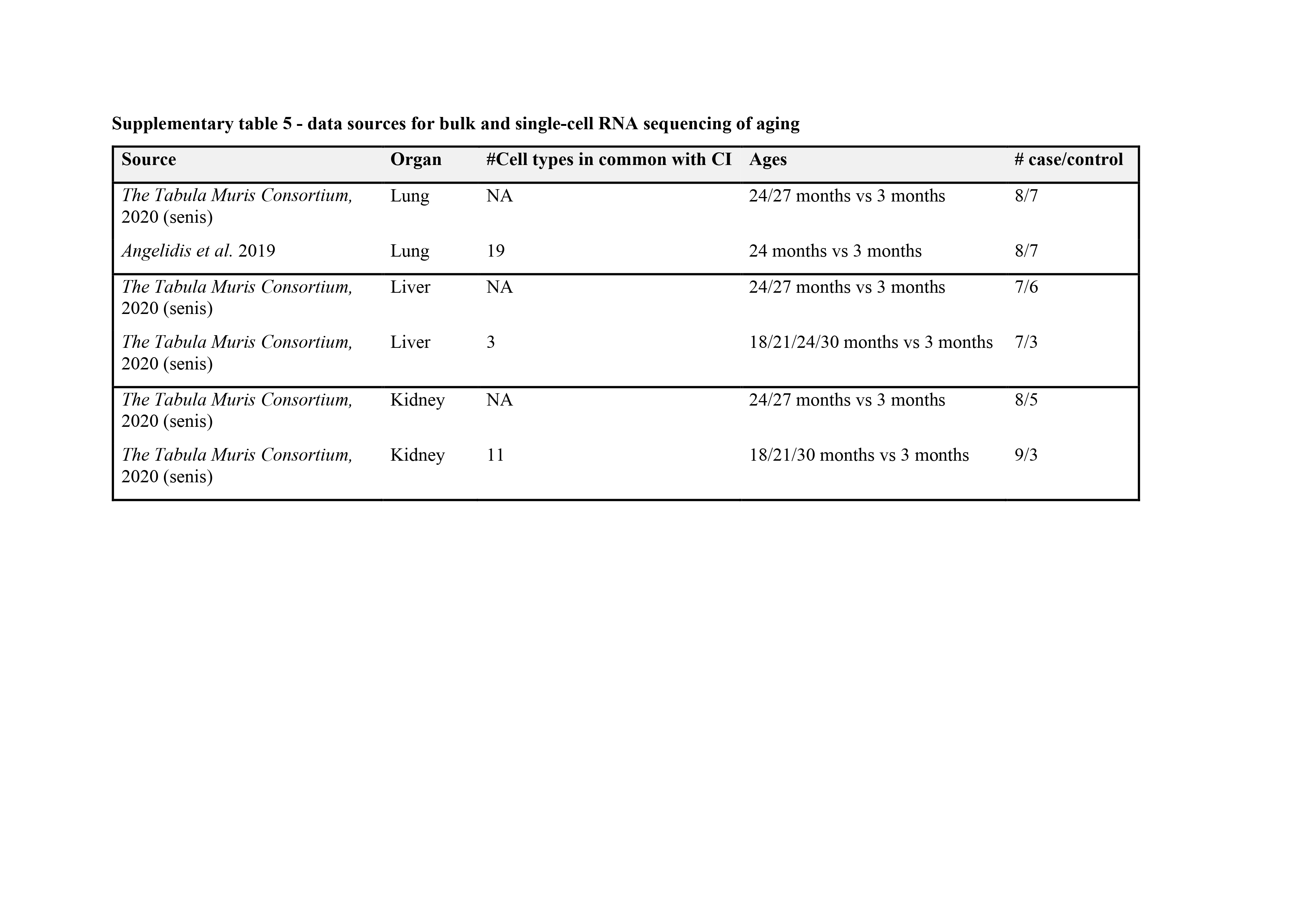

Supplement: Supplementary file 7 — Supplementary file7 (JPG 693 KB) [file 11357_2023_915_MOESM7_ESM.jpg]
